# Management of moderate-to-severe dry eye disease using chitosan-*N*-acetylcysteine (Lacrimera®) eye drops: a retrospective case series

**DOI:** 10.1007/s10792-020-01324-5

**Published:** 2020-03-02

**Authors:** Johannes Nepp, Wolfgang Knoetzl, Anna Prinz, Sonja Hoeller, Martin Prinz

**Affiliations:** 1 Augenzentrum, Musilplatz 9/3, 1160 Vienna, Austria; 2 Department of Ophthalmology, Medical University of Vienna, 1090 Vienna, Austria; 3 Schulstraße 1, 3300 Amstetten, Austria; 4 Rotenturmstraße 1, 1010 Wien, Austria; 5 Clinical Development, CROMA-PHARMA GmbH, Industriezeile 6, 2100 Leobendorf, Austria

**Keywords:** Dry eye disease (DED), Chitosan-*N*-acetylcysteine, Tear film stability, Corneal surface regeneration, Relief of symptoms

## Abstract

**Purpose:**

Dry eye disease is a highly prevalent condition that causes tear film instability, ocular discomfort, and visual disturbance. Lacrimera eye drops are approved for the short-term treatment of dry eye disease. We aimed to evaluate the clinical outcome of patients with moderate-to-severe dry eye disease treated with Lacrimera up to 1 month during routine clinical practice.

**Methods:**

We retrospectively collected data from 25 patients with dry eye disease from the start of Lacrimera treatment up to 1 month of follow-up period. We analyzed standard clinical parameters to follow the course of the patients’ dry eye signs and symptoms.

**Results:**

Based on corneal staining data, we found that the percentage of patients with intact corneas raised from 12 to 64% after 1 month of Lacrimera treatment. During this period, we also observed an increase in both tear breakup time (*p* < 0.05) and Schirmer’s score (*p*  < 0.001), with lower values indicating severer signs. Lacrimera eye drops were judged by 29% of the patients to be effective at relieving eye symptoms.

**Conclusions:**

Lacrimera appears to be safe and effective in the treatment of dry eye disease, as assessed by corneal staining, tear breakup time, and Schirmer’s analyses. Our data suggest that the regenerative effect of Lacrimera eye drops peaks at 2 weeks and is sustained for at least 1 month when administered for a longer period of time.

## Introduction

Dry eye is a multifactorial disease that causes ocular discomfort and visual disturbance [1]. Common eye symptoms include dryness, itching, burning, pressure, or sand corn sensation. Corneal epithelial damage (aka superficial punctate keratopathy) is often observed in patients with severer forms of the disease. Risk factors include age, gender, use of certain medications, and exposure to environmental stressors such as viral infections or polluted air. Dry eye is, indeed, one of the most common reasons for seeking eye care [2]. Dry eye disease is currently regarded as a disorder of the tear film when it fails to cover the entire conjunctiva and cornea and leads to chronic inflammation of the ocular surface [3, 4]. Diagnosis is done based on the patient’s symptoms and the examination of the eye’s ocular surface and tear film.

Topically administered lubricants (artificial tears) are already the mainstay of therapy [5]. However, due to the chronic nature of the disease, ophthalmological formulations with prolonged action are highly needed. Chitosan-*N*-acetylcysteine is a new biopolymer that forms a protective glycocalyx layer on the ocular surface [6]. It is characterized by a 24-h ocular surface retention time [7], which differentiates it from common eye lubricants. Chitosan-*N*-acetylcysteine is derived from chitosan [8, 9], a polycationic linear polysaccharide produced by deacetylation of chitin, which in turn is a structural element present in the exoskeleton of arthropods and cell walls of fungi. The mucoadhesive properties of chitosan are further enhanced by the addition of thiol groups from *N*-acetylcysteine to the chitosan backbone [10]. The beneficial effect of chitosan-*N*-acetylcysteine in ocular wound healing has been attributed to the presence of these thiol groups that facilitate a chemical interaction of the polymer with inner layer mucins, resulting in the stabilization of the polymer–mucin network on the ocular surface [11, 12]. In a rabbit model of corneal debridement, it was shown that the administration of chitosan-*N*-acetylcysteine accelerates corneal healing [6].

Chitosan-*N*-acetylcysteine was introduced in the European market in 2014 as the active compound in eye drops (Lacrimera®). Lacrimera, which is classified as a medical device, is approved for short-term alleviation of dry eye symptoms. A single instillation of Lacrimera significantly increased the median tear film thickness for 24 h [13]. The use of Lacrimera eye drops in moderate-to-severe dry eye patients, which include patients with severer dry eye signs and concomitant diseases, such as Sjögren’s syndrome and glaucoma, has been the focus of a subsequent case series [14]. It was shown that the daily use of Lacrimera for five consecutive days restored the corneal surface and attenuated symptoms in > 80% of patients, as assessed 3 weeks post-treatment. In the present study, we aimed to survey the clinical outcome of patients with moderate-to-severe dry eye disease who were treated with Lacrimera eye drops for > 5 days, taking into account their baseline characteristics.

## Methods

This is a retrospective, longitudinal, uncontrolled case series to evaluate the course of dry eye signs and symptoms in patients treated with Lacrimera eye drops in routine clinical practice.

### Patients

Twenty-five patients suffering from moderate-to-severe dry eye disease [4] of various etiologies who opted to apply Lacrimera for > 5 days were included in this study. Patients’ data were collected retrospectively from three different sites in Austria, namely the ophthalmology clinics of Dr. Knötzl (12 patients), Dr. Nepp (nine patients), and Dr. Prinz (four patients). All patients experienced one or more of the following eye symptoms: dryness, itching, burning, pressure, sand corn sensation, blurred vision, and blepharitis. Moreover, they presented at least one of the following signs: abnormal TBUT < 10 s, abnormal Schirmer’s score < 7 mm (in 5 min), or positive corneal staining. In addition to demographic information and concomitant medication, we gathered patients’ data on dry eye risk factors, including previous eye surgery, history of ocular diseases, rheumatoid arthritis, diabetes mellitus, and thyroid disease.

### Treatment regime

Patients were prescribed Lacrimera eye drops daily for up to 3 months, alone or in combination with other medication.

### Ophthalmological assessments

Follow-up visits were conducted 5 days, 2 weeks, 1 month and 3 months after treatment initiation. The severity of dry eye signs was evaluated by tear breakup time analysis, Schirmer’s test, and corneal fluorescein staining.

Tear breakup time (TBUT) is a reliable assessment of tear film stability, being calculated as the time between the last blink and the appearance of the first dry spot or hole in the tear film. Values under 10 s typically indicate ocular surface dryness.

Schirmer’s test is widely used to examine tear secretion. It is the length (in mm) of wetting a paper strip placed in the inferior cul-de-sac of the lower eyelid in 5 min. Distances shorter than 7 mm indicate an abnormality.

Corneal fluorescein staining is an indicator of corneal surface integrity. The distribution pattern of the dye was assessed using a modified National Eye Institute (NEI) grading system [15].

### Statistics and ethics

For the quantification, we pooled together the data of all patients followed over the same period. The mean, median, standard deviation, and range were used for descriptive purposes. Missing data were not estimated and/or imputed in any way. In case both eyes were affected, only the score of the worse eye was used for the analyses. Incomplete data sets for a specific patient and parameter, i.e., when the patient’s data for that parameter were missing at baseline or 1 month, were excluded from the statistical analysis. The paired Student’s *t* test was used to compare treatment effect before and after medical treatment.

The study was performed in accordance with the Declaration of Helsinki and was approved by the Ethics Committee of Lower Austria.

## Results

A total of 25 patients [age range 24–79, 21 women (84%) and 4 men (16%)] were given Lacrimera as a treatment for dry eye disease of varied etiologies. Among those 25 patients, some patients had also been diagnosed with Sjögren’s syndrome (2/25) or diabetes (1/25), while others had undergone eye surgery (4/25). At the beginning of the study, the average tear breakup time was 4.7 ± 3.2 s and the average Schirmer’s score 7.0 ± 4.8 mm in 5 min. About 85% of patients showed positive corneal staining, indicating ocular surface damage.

The vast majority of the patients reported applying Lacrimera drops once per day at night, although two patients applied it twice per day, in the morning and at night. After 1 month, all except two patients still applied Lacrimera drops daily. All patients reported using concomitant medication, including other lubricant eye drops, gels, and sprays, as well as anti-inflammatory medicine. The top three most used compounds at baseline were sodium hyaluronate (75%), perfluorohexyloctane (31%), and retinol palmitate (25%). While seven out of twenty-five patients (28%) started a new medication during the course of Lacrimera treatment, the large majority (72%) took the same medication at baseline and during Lacrimera treatment.

**Fig. 1:**
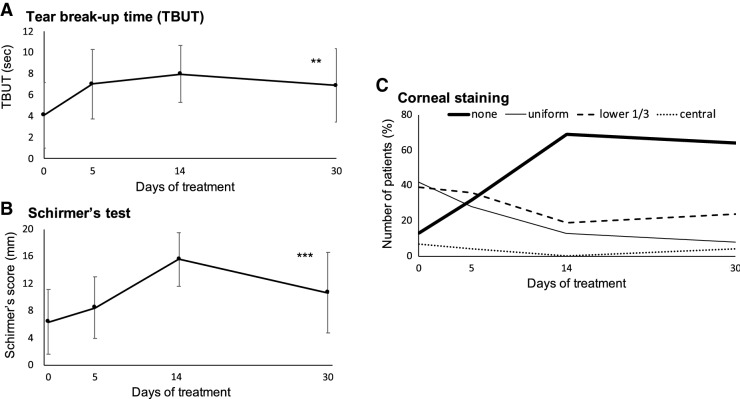
Changes in dry eye signs following Lacrimera treatment. Prolonged treatment with Lacrimera **a** increased tear film stability, **b** promoted tear accumulation, and **c** restored the corneal surface at 1 month in comparison to baseline. The number of patients with intact corneas (“none”) increases following treatment with Lacrimera eye drops. Values are presented as mean ± standard deviation. In case both eyes were affected, only the score with the highest severity was considered. *n* ≥ 18 patients for all clinical parameters at baseline and at 1 month. Statistical significance of the change from baseline was assessed by paired two-tailed Student’s *t* test. TBUT is a measure of tear film stability, with shorter times indicating a greater abnormality. Schirmer’s test evaluates tear secretion, with shorter lengths indicating a greater abnormality. The corneal surface was assessed by fluorescein staining

Treatment with Lacrimera drops increased tear breakup times (4.1 ± 3.1 s at baseline vs 6.9 ± 3.5 s after 1 month; *p* value = 0.0022) and Schirmer’s score (6.4 ± 4.8 mm at baseline vs 10.8 ± 6.0 mm after 1 month; *p* value < 0.001) (Fig. 1a, b). Furthermore, corneal fluorescein staining revealed that Lacrimera treatment promoted the regeneration of the corneal surface. Whereas only 12% of the patients had intact corneas at the beginning of the study, 64% of the patients presented no fluorescein staining after 1 month of treatment (Fig. 1c). The improvement in dry eye signs was accompanied by an improvement in the patients’ symptoms. While all patients stated having one or more dry eye symptoms at the beginning of the study, 29% of the patients were free of symptoms after 1 month of treatment (Table 1). No safety concerns were documented.

**Table 1:** Changes in symptoms of dry eye disease following 1-month Lacrimera treatment

	Baseline	5 days	2 weeks	1 month
Relief of symptoms (% patients without symptoms)	–	1/17 (6%)	4/14 (29%)	5/17 (29%)

Number of patients in whom the clinical symptoms were recorded at each time point. All patients reported symptoms of itching, burning, pressure, sand corn, or blurred vision at the beginning of the study. About 30% of patients said having no symptoms after 1-month Lacrimera treatment.

Ten patients came for the last follow-up visit at 3 months, of which seven were still applying Lacrimera drops daily. Eight patients still showed a beneficial effect of Lacrimera, based on either TBUT (5/10), Schirmer’s test (3/10), or corneal staining scores (intact cornea in 7/10 patients). In two patients, data at 3 months were comparable to baseline values (data not shown).

## Discussion

In dry eye disease, the loss of tear film homeostasis compromises the eye’s ability to maintain the amount of moisture needed to keep the eyes well lubricated and comfortable. Our study provides evidence that the use of Lacrimera eye drops daily for 30 days increases tear film stability and thickness, helping to heal the corneal surface. The improvement in these signs, and a concomitant alleviation of the patients’ symptoms, last for at least 1 month after treatment initiation.

Our corneal staining data showed that the percentage of patients with intact corneas increased from 12% pretreatment to 64% following 1-month Lacrimera treatment. This improvement is clinically meaningful, as the integrity of the corneal surface is a critical factor for proper visual function and optical quality [16]. The wound-healing effect of chitosan-*N*-acetylcysteine has been attributed to its thiol groups, which facilitate the interaction of the polymer with the inner mucin layer [11, 12]. The resulting polymer–mucin network coats the surface of the eye, possibly increasing the ability of the tears to adhere to the corneal epithelial and to protect the ocular surface. This idea is supported by our findings that the mean Schirmer’s score increases by 68% and the mean tear breakup time increases by 50% following Lacrimera treatment for 30 days. This suggests an improvement in tear accumulation and tear film stability, respectively.

Remarkably, Lacrimera seems to have a beneficial effect across varied backgrounds seen in this patient population. The study population includes patients with aqueous-type (65%) and evaporative-type (28%) dry eye disease, patients with a history of eye surgery and Sjögren’s syndrome, among other ailments. Indeed, a major challenge in the treatment of this condition is precisely the discordance between symptoms and the various diagnostic tests such as Schirmer’s test and tear film breakup time. Thus, it suggests that Lacrimera works well in reducing both dry eye signs and symptoms in routine clinical practice. The major limitation of this study is, of course, linked to its retrospective nature. Since only ten patients came to the last follow-up visit at 3 months, while the others may have sought care elsewhere for other treatments, this may suggest a selection bias. That is, we cannot rule out that patients that benefited from treatment are more likely to remain in the study than those who do not benefit from it. Likewise, although patients may feel better after Lacrimera treatment, we cannot neglect a placebo effect or an effect of any of the other concomitant medications.

Our results are consistent with previous reports on the effect of daily use of Lacrimera in dry eye patients. Using other validated grading systems, Messina and Dua showed that the daily use of Lacrimera for five consecutive days improved corneal integrity and attenuated symptoms in > 80% of patients as seen 3 weeks post-treatment [14].

In conclusion, these results provide further support for the extended use of Lacrimera in the management of dry eye symptoms and signs. In this context, it is worth noting the sustained improvement in all the measured parameters over the 1-month treatment period. This suggests that patients may benefit from a more extended period of use of Lacrimera beyond the initial five days of treatment. These results need to be validated in placebo-controlled randomized trials and studies that compare different treatment durations in a larger number of patients.
